# A Cross-Sectional Cohort Study of Extended-Spectrum-Beta-Lactamase-Producing *Enterobacterales* in Patients with Traveler's Diarrhea

**DOI:** 10.1128/AAC.01585-20

**Published:** 2020-11-17

**Authors:** Oskar Ljungquist, Angela Camporeale, Shoeib Nematzadeh, Christian G. Giske, Fredrik Resman, Kristian Riesbeck, Johan Tham

**Affiliations:** a Clinical Microbiology, Department of Translational Medicine, Faculty of Medicine, Lund University, Malmö, Sweden; b Clinical Infection Medicine, Department of Translational Medicine, Faculty of Medicine, Lund University, Malmö, Sweden; c Department of Infectious Diseases, Helsingborg Hospital, Helsingborg, Sweden; d Department of Laboratory Medicine, Karolinska Institute, Stockholm, Sweden; e Department of Clinical Microbiology, Karolinska University Hospital, Stockholm, Sweden

**Keywords:** *E. coli*, ESBL, beta-lactamases, pAmpC

## Abstract

Patients with traveler’s diarrhea (TD) can acquire extended-spectrum-beta-lactamase (ESBL)-producing members of the *Enterobacterales* (EPE) during travel to areas of endemicity. The aim of the present study was to investigate the prevalence and characteristics of EPE carriage in travelers from southern Sweden who were sampled for bacterial diagnostics of TD compared to those of EPE carriage 10 years ago. Clinical samples sent for culture of common causes of bacterial enterocolitis, if the referral stated foreign travel, were included in the study. Antimicrobial susceptibility testing was done according to the EUCAST disk diffusion test method. EPE strains were subjected to whole-genome sequencing (WGS). Eighty-four of 303 patients carried a total of 92 ESBL-producing members of the *Enterobacterales*. The overall prevalence of EPE in tested samples was thus 28%, compared to 24% 10 years earlier (*P = *0.33). Among 86 strains available for WGS, 47 different sequence types (STs) were identified, and there were only 5 ST131 strains. Of the 79 Escherichia coli isolates, 76% carried at least one *fim* (type 1 fimbria) gene, 29% carried at least one *pap* (p-fimbriae) gene, and 43% were extraintestinal pathogenic E. coli (ExPEC) or uropathogenic E. coli (UPEC). Over half of the E. coli strains (57%) were intestinal pathogenic E. coli, most commonly enteroaggregative E. coli (EAEC) (33%), and enteroinvasive E. coli EIEC (22%). A relatively high proportion of patients with traveler’s diarrhea carry EPE, but there was no significant increase compared to 10 years ago. Most E. coli strains were intestinal pathogenic strains. A comparatively high proportion of the strains were ExPEC/UPEC, many expressing the virulence genes *pap* and/or *fim*. (This project was assigned ClinicalTrials.gov number NCT03866291.)

## Abstract (precis)

Patients with traveler’s diarrhea (TD) can acquire extended-spectrum-beta-lactamase (ESBL)-producing members of the *Enterobacterales* (EPE) during travel to areas of endemicity. The aim of the present study was to investigate the prevalence and characteristics of EPE carriage in travelers from southern Sweden who were sampled for bacterial diagnostics of TD compared to those of EPE carriage 10 years ago. Clinical samples sent for culture of common causes of bacterial enterocolitis, if the referral stated foreign travel, were included in the study.

## INTRODUCTION

International travelers, with or without traveler’s diarrhea (TD), frequently carry extended-spectrum-beta-lactamase (ESBL)-producing members of the *Enterobacterales* (EPE) when returning from areas of endemicity (1–10). Sweden has low antimicrobial resistance (AMR) prevalence with regard to the relatively low proportion of EPE found in clinical cultures (11). However, the proportion of *Enterobacterales* with resistance to third-generation cephalosporins is steadily rising worldwide, including in Sweden (11–15). A fecal culture positive for EPE is a major risk factor for EPE bloodstream infection, requiring treatment with broad-spectrum antibiotics, such as carbapenems (16).

In worldwide travelers, common etiologies of traveler’s diarrhea include enteropathogenic Escherichia coli (EPEC), enteroaggregative E. coli (EAEC), enterotoxigenic E. coli (ETEC), Campylobacter jejuni, *Salmonella* spp., and *Shigella* spp. (17, 18). Of these, all but C. jejuni have been demonstrated to carry genes encoding ESBL on plasmids or on the chromosome.

A decade ago, we found high prevalence (24%) of EPE in fecal cultures from international travelers returning to southern Sweden with traveler’s diarrhea (1). Owing to the worldwide rise in EPE prevalence, we hypothesized that the prevalence of EPE in patients with TD had increased during this decade. The primary aim of the present investigation was thus to repeat the previous study from 2010 with similar methods to compare the prevalence of EPE carriage. The null hypothesis was that the prevalence of EPE had not increased during the last decade. Furthermore, we wanted to characterize the identified strains using molecular genetic methods. Secondary aims were therefore to characterize the EPE isolates phenotypically and study sequence type, resistance, and virulence genes using whole-genome sequencing. We wanted to investigate the enteric pathotype of E. coli and the prevalence of carbapenem-resistant strains and to examine species, phenotypes, and susceptibility to clinically important antibiotics.

## RESULTS

### International travel and ESBL acquisition.

At the Department of Clinical Microbiology of Skåne University Hospital, 314 samples were subjected to selective EPE culture. For 11 of these, the referral stated “asymptomatic.” Of 303 Swedish patients with traveler’s diarrhea, 84 (28%) carried a total of 92 ESBL-producing strains of *Enterobacterales*.

Of the 303 patients, 124 (41%) were men and 179 (59%) women, and the median age of all patients was 46 (range, 1 to 90 years).

Travel destinations (by continent) were Europe excluding Sweden (36%), Asia (33%), Africa (18%), North America and the Caribbean (6%), not specified (5%), South and Central America (3%), and Oceania (0%). EPE prevalence was highest for travelers from Africa (54%), Asia (45%), and North America and the Caribbean (22%). EPE prevalences with respect to specific regions and selected countries are displayed in Table 1. An extensive list of countries visited by travelers is presented in Table S1.

**TABLE 1:** Proportion of EPE-positive samples with respect to region

Location	No. of samples			% positive	95% CI^*a*^ (%)	*P*^*b*^	Adjusted significance^*c*^
	EPE positive	EPE negative	Total				
World	84	219	303	28	23–33		

Continent							
Asia	45	54	99	45	35–56	<0.0001	Significant
Africa	28	24	52	54	40–68	<0.0001	Significant
South and Central America	1	8	9	11	0–48	0.45	Not significant
North America and Caribbean	4	14	18	22	6–48	0.79	Not significant
Europe^*d*^	5	104	109	5	2–10	<0.0001	Significant^*e*^

Country/region							
Egypt	5	2	7	71	29–96	0.02	Not significant
Thailand	9	17	26	35	17–0.56	0.49	Not significant
Turkey	7	9	16	44	20–70	0.16	Not significant
India	8	0	8	100	63–100	<0.0001	Significant
Kenya, Tanzania (including Zanzibar)	8	2	10	80	44–98	0.0007	Significant
South Africa	5	5	10	50	19–81	0.15	Not significant
Cuba	4	3	7	57	18–90	0.10	Not significant
Unspecified	1	14	15	7	0–32	0.08	Not significant

a CI, confidence interval.

b Compared to the world value (excluding Sweden and the country/region compared).

c Adjusted according to the Bonferroni correction: 0.05/(*n *= 13) = 0.0038, where *n* is number of hypotheses.

d Excluding Sweden.

e Note the significantly lower prevalence of EPE in travelers to Europe.

Of the 92 EPE strains, 92% were E. coli and the remaining 8% were Klebsiella pneumoniae. Furthermore, 9 strains were AmpC producing, of which 4 expressed phenotypic inhibition by both cloxacillin and clavulanic acid; the remaining 85 were inhibited solely by clavulanic acid (ESBL).

### Susceptibility testing.

**FIG 1:**
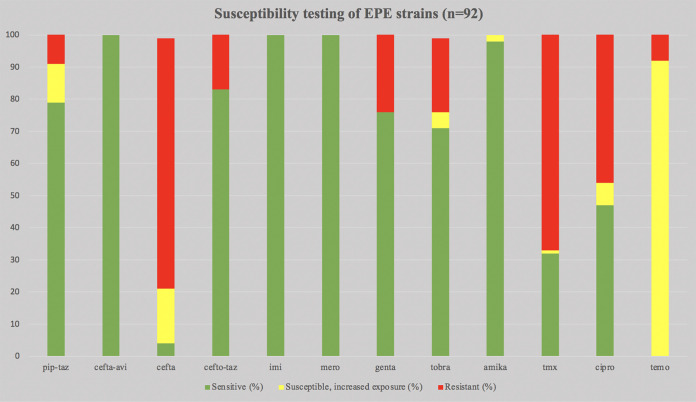
Susceptibility testing of EPE strains (*n* = 92). The percentages of strains classified as susceptible; susceptible, increased exposure; and resistant are shown. Abbreviations: pip-taz, piperacillin-tazobactam; cefta-avi, ceftazidime-avibactam; cefta, ceftazidime; cefto-taz, ceftolozane tazobactam; imi, imipenem; mero, meropenem; genta, gentamicin; tobm, tobramycin; amika, amikacin; tmx, trimethoprim-sulfamethoxazole; cipro, ciprofloxacin; temo, temocillin. All percentages were rounded off to the nearest integer.

All 92 EPE strains in the study were susceptible to meropenem, imipenem, and ceftazidime-avibactam. “Susceptible, increased exposure” was found for piperacillin-tazobactam (12%), ceftazidime (17%), tobramycin (%), amikacin (2%), trimethoprim-sulfamethoxazole (1%), ciprofloxacin (7%), and temocillin (92%). Resistance was found for piperacillin-tazobactam (9%), ceftazidime (78%), ceftolozane-tazobactam (17%), gentamicin (24%), tobramycin (23%), trimethoprim-sulfamethoxazole (67%), ciprofloxacin (47%), and temocillin (8%). The results are summarized in Fig. 1.

### Results of screening for *Campylobacter*, *Salmonella*, *Yersinia*, and *Shigella*.

There was no difference in proportions of patients positive for *Campylobacter*, *Salmonella*, *Yersinia*, or *Shigella* between the EPE-colonized (5/84; 6.0%) and EPE-negative (13/219; 5.9%) (*P = *1) groups.

### Sequence types and phylogroup.

Among 86 EPE strains (79 E. coli and 7 K. pneumoniae) available for whole-genome sequencing, 47 different sequence types were identified, of which 3 were novel. The most common were ST38 (*n *= 9; 11%) and ST10 (*n *= 7; 9%); only 5 strains (6%) belonged to ST131.

Most E. coli isolates belonged to phylogroup A (*n *= 32; 41%) or D (*n *= 25; 32%). Less prevalent were B2 (*n *= 8; 10%), B1 (*n *= 4; 5%), and F (*n *= 2; 3%). Sequence types are displayed in Table S2, and a complete list of phylogroups is displayed in Table S4.

### E. coli pathotypes and virulence genes.

For more than half of the EPE strains (*n *= 45; 57%), an intestinal pathogenic pathotype was found, with EAEC (*n *= 26; 33%) and EIEC (*n *= 17; 22%) being most prevalent. One ETEC strain, one EPEC strain, and no EHEC strains were found. For 27 isolates, no enteric pathotype could be determined.

Furthermore, 34 strains (43%) were either ExPEC or UPEC, and of these, 24 strains (30%) carried genes shared by ExPEC/UPEC and EAEC or EIEC. There was no statistical difference in the risk of carrying an ExPEC/UPEC strain and travel destination (region). Carriage of ExPEC/UPEC strains and travel destinations are displayed in Table S3.

Of the 79 E. coli isolates, 60 (76%) strains carried at least one type 1 fimbria gene and 59 (75%) carried at least two. Furthermore, at least 24 (30%) strains carried at least one *pap* (p-fimbria) gene, and 21 (27%) carried at least two. Virulence genes and geographical distributions are displayed in Table 2.

**TABLE 2:** Bacterial characteristics in relation to region

Characteristic	No. (%) of isolates from region								*P*^*b*^
	World^*a*^	Asia	Africa	South and Central America	North America and Caribbean	Europe	Unspecified	Total	
Beta-lactamase									
CTX-M-15	53 (91)	29 (50)	22 (38)	0 (0)	2 (3)	4 (7)	1 (2)	58 (100)	0.2911
CTX-M-27	9 (100)	6 (67)	1 (11)	1 (11)	1 (11)	0 (0)	0 (0)	9 (100)	0.2387
CTX-M-14	7 (100)	4 (57)	3 (43)	0 (0)	0 (0)	0 (0)	0 (0)	7 (100)	1
CTX-M-55	3 (100)	3 (100)	0 (0)	0 (0)	0 (0)	0 (0)	0 (0)	3 (100)	0.28

Phylogroup									
A	27 (96)	18 (64)	8 (29)	0 (0)	1 (4)	1 (4)	0	28 (100)	0.45
D	22 (88)	9 (36%)	10 (40)	1 (4)	2 (8)	3 (12)	0	25 (100)	0.17

Virulence factor in E. coli									
*pap*	19 (86)	12 (55)	7 (32)	0 (0)	0 (0)	3 (14)	0 (0)	22 (100)	1
*fim*	51 (89)	30 (53)	17 (30)	0 (0)	4 (7)	5 (9)	1 (2)	57 (100)	0.62
ExPEC/UPEC	28 (88)	15 (47)	11 (34)	1 (3)	1 (3)	4 (13)	0 (0)	32 (100)	0.62

Resistance genes									
Fluoroquinolones	34 (83)	23 (56)	8 (20)	0 (0)	3 (7)	6 (14)	1 (2)	41 (100)	0.09
Aminoglycosides	58 (89)	33 (51)	21 (32)	1 (2)	3 (5)	7 (11)	0 (0)	65 (100)	1
Trimethoprim	56 (88)	32 (50)	20 (31)	1 (2)	3 (5)	8 (13)	0 (0)	64 (100)	1
Sulfonamide	58 (91)	33 (52)	21 (33)	1 (2)	3 (5)	6 (9)	0 (0)	64 (100)	1
Fosfomycin	6 (100)	3 (50)	3 (50)	0 (0)	0 (0)	0 (0)	0 (0)	6 (100)	0.67
Aminoglycosides and fluoroquinolones (combined)	10 (100)	6 (60)	4 (40)	0 (0)	0 (0)	0 (0)	0 (0)	10 (100)	1

a Excluding Europe and unspecified.

b Univariate analysis compared only Asia and Africa in the analysis. Four patients carried two EPE strains, but only one strain was included in the analysis.

### Resistance genes.

In total, 145 different beta-lactam resistance genes were identified. ESBL genes found were *bla*_CTX-M-15_ (*n *= 62), *bla*_CTX-M-27_ (*n *= 9), *bla*_CTX-M-14_ (*n *= 7), and *bla*_CTX-M-55_ (*n *= 3). pAmpC genes found were *bla*_DHA-1_ (*n *= 4), *bla*_CMY-2_ (*n *= 3), and *bla*_CMY-42_ (*n *= 2). Other beta-lactamase genes found were *bla*_TEM-1B_ (*n *= 29), *bla*_OXA-1_ (*n *= 11), *bla*_TEM-35_ (*n *= 4), *bla*_SHV-199_ (*n *= 2), *bla*_SHV-27_ (*n *= 2), *bla*_SHV-106_ (*n *= 1), *bla*_SHV-60_ (*n *= 1), *bla*_TEM-1C_ (*n *= 1), *bla*_SHV-159_ (*n *= 1), *bla*_OKP-B-12_ (*n *= 1), *bla*_OKP-B-14_ (*n *= 1), and *bla*_OKP-B-3_ (*n *= 1).

Of the 86 genotyped EPE strains, 79% carried genes for resistance against aminoglycosides, 51% for fluoroquinolones, 77% for sulfonamides, 78% for trimethoprim, 7% for fosfomycin, 24% for chloramphenicol, and 12% for aminoglycosides and fluoroquinolones (combined).

Genotypic and phenotypic expression of AmpC correlated well (*n *= 9). Resistance genes and geographical distributions are displayed in Table 2.

## DISCUSSION

The primary aim of this study was to investigate the EPE prevalence in patients with traveler’s diarrhea in the south of Sweden following international travel. The prevalence of 28% was slightly higher than in an investigation in a similar population 1 decade earlier (24%), but the difference was not statistically significant (1). There was no difference in travel destinations between the two studies that could influence this, with similar odds ratios for EPE prevalence with respect to continents (Table S5).

The method and the types of patients included were very similar in both studies. During this decade, international travel more than doubled worldwide, and European data show a rising prevalence of EPE in clinical samples (11, 19). Despite these changes, we did not observe an increased prevalence of EPE in patients with traveler’s diarrhea.

The EPE prevalence of 28% is comparable to that in other studies of international travelers (3, 6, 10, 20–22). Similar to prior travel studies, EPE prevalence in relation to region visited was highest for Africa (54%) and Asia (45%) (13). In contrast, EPE prevalence was significantly lower for Europe (5%) compared to the rest of the world, which is comparable to the EPE prevalence in healthy Swedish individuals (23).

Despite a small number of patients, the EPE prevalence in travelers from India (100%), Kenya, Tanzania including Zanzibar (80%), and Egypt (71%) was remarkably high.

Regarding secondary aims, no strains were resistant to carbapenems, ceftazidime-avibactam, or amikacin. Ceftazidime-avibactam and ceftolozane-tazobactam are recently introduced antibiotics and may thus be promising carbapenem-sparing alternatives for treating severe EPE infections. While no ceftazidime-avibactam-resistant strains were found, 17% of the strains were resistant to ceftolozane-tazobactam. Such a high resistance rate could advise against the empirical usage of ceftolozane-tazobactam in the clinical setting, at least in countries where EPE is highly endemic.

It is interesting that while ESBL and AmpC resistance genes are fully expressed phenotypically, genotypical and phenotypical resistance did not entirely correspond for aminoglycosides, fluoroquinolones, and trimethoprim-sulfamethoxazole. However, genes encoding aminoglycoside resistance do not always affect susceptibility to gentamicin, tobramycin, or amikacin. Alternatively, this could be the result of plasmid acquisition, with the genes staying unexpressed.

Our study revealed a heterogeneous group of sequence types in our cohort of travelers, three of which were not previously reported. Only 5 (6%) strains belonging to the pandemic high-risk clone ST131 were found, which is lower than in earlier studies (10, 24). This is somewhat surprising, considering the rather high proportion of strains (43%) with the potential to cause extraintestinal infections. This proportion of ExPEC was similar to a recent study in Finland (25). Furthermore, the rate of fimbria-encoding virulence genes found in these EPE strains was comparable to that in a recent Swedish study, as was strain distribution according to phylogroup (10). However, compared to that study, a considerably higher proportion of pyelonephritis-associated pilus-encoding genes were found in our study (30% versus 9.2%). There were no significant differences between strains from Africa and Asia with respect to virulence or resistance genes. As our cohort consisted solely of patients with diarrhea, it is not surprising that the majority of E. coli strains found were diarrheagenic E. coli. More surprising is the relatively high prevalence of virulence factors found.

Acquisition of virulent EPE strains during travel is common, but the risk of EPE infections acquired during travel has not been studied satisfactorily. Further studies on the implication and spread of EPE carriage after international travel is warranted.

Our cohort was not cultured for EPE before traveling, which is a major limitation of the study. This prevented us from detecting the true EPE acquisition rate. Several years ago, a Swedish study of community carriage of EPE in Sweden found a colonization rate of 5% (23). Assuming this colonization rate to be stable, approximately 15 travelers could have been EPE positive prior to their trip. A further limitation is lack of data on severity of diarrhea, length of travel, and patient characteristics.

Furthermore, only one culture was used to determine the presence of EPE. As the sensitivity of detecting EPE increases for each culture obtained, the true prevalence of EPE could be higher. Additionally, the method of relying solely on information from microbiological referrals risks performance bias. This approach was not validated prior to the study.

A strength of this study is the inclusion of a sizable number of travelers to a broad diversity of international destinations. In contrast to many other travel studies, this study included travelers regardless of whether pretravel consultation was needed. This decreases the bias that a pretravel culture could give rise to, which is often limited to individuals who seek pretravel medical advice. Furthermore, this cohort includes only patients with diarrhea, a group which has not been studied extensively previously.

Further prospective studies should aim to explore the risks of clinical infections and the spread and duration of posttravel EPE colonization, preferably obtaining pretravel cultures and multiple posttravel cultures. It would be interesting to investigate if prolonged EPE carriage and risk of infection and spread could be associated with specific virulence traits.

### Conclusion.

We performed a cross-sectional cohort study in south Sweden. A relatively high proportion (28%) of patients with traveler’s diarrhea carried EPE with a broad diversity of sequence types. This is a slight but not statistically significant increase over the same statistic 10 years before (28% versus 24%). Most E. coli strains were intestinal pathogenic E. coli. A comparatively high proportion of the strains were ExPEC/UPEC, many expressing the virulence genes *pap* and/or *fim*.

## MATERIALS AND METHODS

### Study setting.

The Department of Clinical Microbiology, Skåne University Hospital, serves a population of 1.4 million inhabitants (26). Clinical samples sent to the department for culture of *Campylobacter*, *Salmonella*, *Yersinia*, or *Shigella*, only if the referral stated foreign travel, were included between 23 February 2017 and 9 January 2018. The proportion of included cases varied during the sampling period, but this was mostly due to staff adherence to the study protocol, and it is not expected that this introduced a systematic bias. Another source of bias is that clinicians do not always include information regarding travel on the referral. It is impossible to determine the exact proportion of cases that were captured, but no systematic selection was done in the microbiology laboratory. Information about countries visited was extracted from the microbiological referrals. These samples were selectively cultivated for EPE according to the hospital protocols. If there were multiple clinical samples from the same patient, only one culture with a positive ESBL (the first) was selected for the study.

### Microbiology.

Briefly, the sample material was plated on URI-Select agar plates with vancomycin (Bio-Rad, Hercules, CA, USA) and on ChromID ESBL chromogenic agar plates (bioMérieux, Marcy-l’Étoile, France) and incubated at 37°C overnight. Two antimicrobial susceptibility discs containing ceftazidime (10 μg/ml; Oxoid, Basingstoke, UK) and meropenem (10 μg/ml; Oxoid) were added to the URI-Select agar plates. Colonies of presumptive EPE were subcultivated on horse blood agar (HBA) or URI-Select agar and typed to bacterial species using matrix-assisted laser desorption ionization–time of flight (MALDI-TOF; Bruker Daltonics, Bremen, Germany).

The phenotype of EPE was characterized by susceptibility to cloxacillin (AmpC) or clavulanic acid (ESBL) using the MAST test (Mast Group Ltd., Liverpool, England). All EPE strains were tested for susceptibility against the following antimicrobial agents: piperacillin-tazobactam, cefotaxime, ceftazidime-avibactam, ceftazidime, ceftolozane-tazobactam, imipenem, meropenem, gentamicin, tobramycin, amikacin, trimethoprim-sulfamethoxazole, ciprofloxacin, and temocillin.

### Whole-genome sequencing.

The EPE strains were investigated using whole-genome sequencing. In brief, DNA extraction was done with the EZ1 Advanced XL system (Qiagen, Hilden, Germany). The quantity of the extracted DNA was measured using a Qubit double-stranded-DNA (dsDNA) assay kit (Life Technologies Europe, Bleiswijk, Netherlands). Extracted DNA was sequenced on Illumina HiSeq 2500 at the Science for Life Laboratory (SciLifeLab, Solna, Sweden), All isolates had a mean sequencing depth of >30×. Assembly of raw reads was obtained via Unicycler (27). The identification of resistance and virulence genes was performed through the in-house bioinformatic pipeline microSALT (https://github.com/Clinical-Genomics/microSALT). An additional pool of virulence genes was retrieved from the VirulenceFinder 2.0 web tool (28).

Enteric pathotype assignment was based on the VFDB (Virulence Factors of Pathogenic Bacteria) database (29). ExPEC was defined as the presence of two or more of the virulence genes *pap*, *sfa/foc*, *afa/dra*, *kpsMT*, and *iutA* (30). UPEC was defined as the presence of three or more of the genes *chuA*, *fyuA*, *vat*, and *yfcV* (31). Phylogroup determination was executed by the ClermonTyping 1.4.0 web tool (32).

### Ethics and Clinicaltrials.gov.

This study was granted ethical approval from the Regional Ethical Review Board at Lund’s District Court (Swedish Ethical Review Authority) with reference number 2016-740. All patients included in the study were contacted by mail, informed about the study, counselled on EPE. This project was assigned ClinicalTrials.gov number NCT03866291.

### Statistics.

Continuous data were summarized as medians and categorical data as proportions. Statistical differences in categorical data were analyzed using Fisher’s exact test. A *P* value of <0.05 was considered statistically significant. Due to multiple comparisons, the Bonferroni correction method was used. The statistical analyses were performed using IBM SPSS Statistics for Macintosh, version 25.0 (IBM, Armonk, NY).

## Supplementary Material

Supplemental file 1
