# Supplementary material for: A Cross-Sectional Cohort Study of Extended-Spectrum-Beta-Lactamase-Producing Enterobacterales in Patients with Traveler's Diarrhea
Source: Antimicrob Agents Chemother. 2020 Nov 17;64(12):e01585-20. doi: 10.1128/AAC.01585-20 (PMC7674057; doi:10.1128/AAC.01585-20)
Supplement: Supplemental file 1 [file AAC.01585-20-s0001.pdf]

Supplementary material.

Table S1. EPE strain according to species, phenotype and countries visited.

Patient	EPE culture	Country/region visited
1	*E. coli* ESBL type A	Dubai
2	*E. coli* ESBL type A	South America
3	*E. coli* ESBL type A, two strains	India
	K. pneumoniae ESBL type A	Thailand
5	*E. coli* ESBL type A	Cuba
6	*E. coli* ESBL type A	Tanzania
7	*K. pneumoniae* ESBL type A	South Africa
8	*E. coli* ESBL type A, two strains	Egypt
9	*E. coli* ESBL-type M (AmpC)	Namibia
10	*E. coli* ESBL type A	Egypt
11	*E. coli* ESBL type A, two strains	Thailand
12	*E. coli* ESBL type A and *K. pneumoniae* ESBL type A	Egypt
13	*E. coli* ESBL type A	Asia
14	*E. coli* ESBL type A	Cambodia
15	*E. coli* ESBL type A	Tanzania
16	*E. coli* ESBL type A	Kenya & Zanzibar
17	*E. coli* ESBL type A	India
18	*E. coli* ESBL type A	Egypt
19	*E. coli* ESBL type A	Thailand
20	*E. coli* ESBL type A	South Africa
21	*E. coli* ESBL type A	Bali
22	*E. coli* ESBL type A	South Africa
23	*E. coli* ESBL-type M (AmpC)	Thailand
24	*E. coli* ESBL type A	India
25	*E. coli* ESBL type A	South Africa
26	*E. coli* ESBL type A	Egypt
27	*E. coli* ESBL type A	Goa, India
28	*E. coli* ESBL type A	India
29	*E. coli* ESBL type A	Thailand

30	*E. coli* ESBL type A	Hungary
31	*E. coli* ESBL type A	Afghanistan
32	*E. coli* ESBL type A and ESBL-M, one strain	Vietnam
33	*E. coli* ESBL type A	Not specified
34	*E. coli* ESBL type A	South Africa
35	*E. coli* ESBL type A	Lebanon
36	*E. coli* ESBL type A	Hungary
37	*E. coli* ESBL type A	Canary Islands
38	*E. coli* ESBL type A	Afghanistan
39	*E. coli* ESBL type A	Not specified
40	*E. coli* ESBL type A	Thailand
41	*E. coli* ESBL type A	Thailand
42	*E. coli* ESBL type A	Turkey
43	*E. coli* ESBL type A	Tunisia
44	*E. coli* ESBL type A	Kenya
45	*E. coli* ESBL type A	Turkey
46	*K. pneumoniae* ESBL type A+ *E. coli* ESBL type A	Sierra Leone
47	*E. coli* ESBL type A	Indonesia
48	*E. coli* ESBL type A	Turkey
49	*E. coli* ESBL type A	Cyprus
50	*E. coli* ESBL type A	Eritrea
51	*E. coli* ESBL type A	Iraq
52	*E. coli* ESBL type A	Tanzania
53	*E. coli* ESBL type A	Mongolia
54	*E. coli* ESBL type A	Armenia
55	*E. coli* ESBL type A	Lebanon
56	*E. coli* ESBL type A	Iraq
57	*E. coli* ESBL type A	Greece
58	*E. coli* ESBL type A	Thailand
59	*E. coli* ESBL type A	Cyprus

60	*E. coli* ESBL type A	Russia
61	*E. coli* ESBL type A	Turkey
62	*E. coli* ESBL-type M (AmpC)	Turkiey
63	*E. coli* ESBL type A and *K. pneumoniae* ESBL type A	India
64	*E. coli* ESBL type A	Turkey
65	*E. coli* ESBL type A	Iran
66	*E. coli* ESBL type A	Africa
67	*E. coli* ESBL type A	Egypt
68	*E. coli* ESBL type A	India
69	*E. coli* ESBL type A	China
70	*E. coli* ESBL-type M (AmpC)	England
71	*E. coli* ESBL type A	Not specified
72	*K. pneumoniae* ESBL type A	Morocco
73	*E. coli* ESBL type A and type M(AmpC), one strain	Turkey
74	*E. coli* ESBL type and type M(AmpC), both in two strains	Thailand
75	*E. coli* ESBL type A and *K. pneumoniae* ESBL type A	Spain
76	*E. coli* ESBL type A	Zanzibar
77	*E. coli* ESBL type A, two strains	Kenya
78	*E. coli* ESBL type A	Cuba
79	*E. coli* ESBL type A	Cuba
80	*E. coli* ESBL type A	Iraq
81	*E. coli* ESBL-type M (AmpC)	Cuba
82	*E. coli* ESBL type A	China, Vietnam
83	*E. coli* ESBL type A	India
84	*E. coli* ESBL type A	Africa

Table S2. Distribution of sequence types.

**ST**	***n***
38	9
10	7
167	6
131	5
48	4
394	4
69	3
405	3
Novel	3
43	2

450	2
34	2
648	2
315	1
120	1
636	1
117	1
219	2
95	1
4	1
8149	1
225	1
31	1
130	1
6448	1
1303	1
73	1
2040	1
15	1
295	1
2797	1
37	1
2332	1
432	1
540	1
58	1
414	1
3045	1

3036	1
88	1
5041	1
1177	1
2325	1
44	1
208	1
40	1
484	1

Table S3. Carriage of ExPEC/UPEC according to continent.

Continent	*n*	ExPEC/UPEC	other	*p* value
Asia	43	16	27	0.35
Africa	22	12	10	0.21
North America	4	1	3	0.63
Europe	4	2	2	1
South America	1	1	0	0.43
Not specified	1	0	1	1
Total	75	32	43	

Table S4. Phylogroups of the 80 *E. coli* strains.

Phylogroup	*n*
A	32
B1	4
B2	8

A/B1	2
Non Esch/B1	1
C	1
D	26
F	2
E	1
E/D	1
D/B1	1
Unknown/F	1

Table S5. Comparing travel destinations in the present study with Tham et al(1).

Present study	EPE positive	EPE negative	EPE rate (%)	Odds ratio*	Tham et al.	EPE positive	EPE negative	EPE rate (%)	Odds ratio*	p-value
Europe	5	104	5	1	Europe	2	61	3	1	0.65
World	84	219	28	6	World	58	184	24	8	0.32
Africa	28	24	54	12	Africa	21	34	38	12	0.1
Asia	45	54	45	10	Asia	28	45	38	12	0.35
America	5	22	18.5	4	America	1	9	10	3	0.53
Unspecified	1	14	7	-	Unspecified	5	36	12	-	0.55

*rounded to the nearest integer.

References

1. Tham J, Odenholt I, Walder M, Brolund A, Ahl J, Melander E. 2010. Extended-spectrum beta-lactamase-producing Escherichia coli in patients with travellers' diarrhoea. Scand J Infect Dis 42:275-80.
